# Deep Intraclonal Analysis for the Development of Vaccines against Drug-Resistant *Klebsiella pneumoniae* Lineages

**DOI:** 10.3390/ijms25189837

**Published:** 2024-09-11

**Authors:** Ana Tajuelo, Eva Gato, Jesús Oteo-Iglesias, María Pérez-Vázquez, Michael J. McConnell, Antonio J. Martín-Galiano, Astrid Pérez

**Affiliations:** 1 Intrahospital Infections Unit, National Centre for Microbiology, Instituto de Salud Carlos III (ISCIII), Majadahonda, 28220 Madrid, Spain; a.tajuelomp@isciii.es (A.T.); eva.gato@isciii.es (E.G.); astrid.perez@isciii.es (A.P.); 2 Universidad Nacional de Educación a Distancia (UNED), 28015 Madrid, Spain; 3 Reference and Research Laboratory for Antibiotic Resistance and Health Care Infections, National Centre for Microbiology, Instituto de Salud Carlos III (ISCIII), Majadahonda, 28220 Madrid, Spain; jesus.oteo@isciii.es (J.O.-I.); mperezv@isciii.es (M.P.-V.); 4 CIBER de Enfermedades Infecciosas (CIBERINFEC), Instituto de Salud Carlos III (ISCIII), 28029 Madrid, Spain; 5 Department of Biological Sciences, University of Notre Dame, Notre Dame, IN 46556, USA; mcconnell.mike75@gmail.com; 6 Core Scientific and Technical Units, Instituto de Salud Carlos III (ISCIII), Majadahonda, 28220 Madrid, Spain

**Keywords:** antibiotic resistance, betweenness centrality, epitope, immunoinformatics, intrahospital infection, natural processing language, neutralizing antibody, protein hub, virulence factor, reverse vaccinology

## Abstract

Despite its medical relevance, there is no commercial vaccine that protects the population at risk from multidrug-resistant (MDR) *Klebsiella pneumoniae* infections. The availability of massive omic data and novel algorithms may improve antigen selection to develop effective prophylactic strategies. Up to 133 exposed proteins in the core proteomes, between 516 and 8666 genome samples, of the six most relevant MDR clonal groups (CGs) carried conserved B-cell epitopes, suggesting minimized future evasion if utilized for vaccination. Antigens showed a range of epitopicity, functional constraints, and potential side effects. Eleven antigens, including three sugar porins, were represented in all MDR-CGs, constitutively expressed, and showed limited reactivity with gut microbiota. Some of these antigens had important interactomic interactions and may elicit adhesion-neutralizing antibodies. Synergistic bivalent to pentavalent combinations that address expression conditions, interactome location, virulence activities, and clone-specific proteins may overcome the limiting protection of univalent vaccines. The combination of five central antigens accounted for 41% of all non-redundant interacting partners of the antigen dataset. Specific antigen mixtures represented in a few or just one MDR-CG further reduced the chance of microbiota interference. Rational antigen selection schemes facilitate the design of high-coverage and “magic bullet” multivalent vaccines against recalcitrant *K. pneumoniae* lineages.

## 1. Introduction

Rationally-designed prophylactic strategies have been underutilized against difficult-to-treat pathogens [1,2]. One of these microorganisms is *Klebsiella pneumoniae*, a gram-negative bacterium found in the environment as well as in the gastrointestinal and respiratory tract microbiota of animals and humans [3]. *K. pneumoniae* can cause a range of life-threatening infections, such as sepsis, pneumonia, or meningitis, as well as urinary tract and wound infections. The two most worrisome epidemiological behaviors observed in this pathogen comprise the hypervirulent and the multidrug-resistant (MDR) phenotypes [4]. The former affects young immunocompetent individuals and are usually susceptible to antibiotics [5]. In contrast, the latter, or classical phenotype, involves MDR lineages that cause worldwide opportunistic infections in elderly, immunocompromised, and hospitalized patients [6,7,8,9]. MDR *K. pneumoniae* clones are most responsible for increased morbidity and mortality attributed to this species. These are currently estimated to cause a third of intrahospital infections [10]. These lineages, mainly those that are carbapenem-resistant, can cause trans-border outbreaks and are considered critical pathogens by the World Health Organization, for which new treatments are urgently needed [11].

Vaccination may be an effective alternative to conventional chemotherapy for preventing *K. pneumoniae* infections, given the challenges associated with developing new antibiotics. Vaccination use could decrease antimicrobial resistance in several ways, including reduction of carriage, bacterial load, and antibiotic use [12]. However, B-cell epitopes of pathogen antigens often undergo strong selective pressure through mutation, recombination, or deletion to evade the host’s immune response [13]. Strategies to develop an effective vaccine against *K. pneumoniae*, including attenuated and inactivated vaccines, outer membrane vesicles, proteins, polysaccharides, and conjugated vaccines, were rather unsuccessful [14,15]. Reasons for failure included lipopolysaccharide toxicity and limited coverage of the 24-valent capsular vaccine. The most advanced prototype is the tetravalent KlebV4 vaccine (identifier: NCT04959344), which is in phase 1/2 trial. As a result, *K. pneumoniae* remains classified into “Group C: pathogens with vaccine candidates either in early clinical trials or with moderate to high feasibility of vaccine development” [16]. This modest biotechnological status, despite the medical relevance of these infections, highlights the need for continued development of novel strategies for combating *K. pneumoniae*.

The limited protection achieved by antigens selected by traditional approaches has led to the use of “omics”-based approaches for antigen identification. On the one hand, experimental techniques such as immunoproteomics have detected real antigens based on patient antisera samples using proteomic tools [17,18,19]. On the other hand, knowledge-based tools such as reverse vaccinology (RV) use pre-existing data and algorithms to potentially outperform reductionist approaches [20]. RV aims to identify the most protective antigens from complete pathogen genomes after the application of selection criteria that include the presence of epitopes by immunoinformatic tools, sequence conservation, non-homology with human proteins, exposure to the immune system, experimental workability, and functional importance. Protocols have been enriched with a finer knowledge of human immunity as well as further post-genomic criteria involving massive genome utilization and protein structural information [21,22]. Compared to empirical strategies, RV presents advantages such as a major control on the immunological response, the inclusion of whole proteomes in the analysis, the facilitation of updating the vaccine if needed, and savings in terms of labor, cost, and time [20]. Data and algorithmic advances promise to revolutionize antigen selection for vaccines. For example, the availability of tens of thousands of genomic sequences per pathogen enables deep intra-clonal search for epitope drift in lineages of interest. Omic-level expression information allows for the identification of important antigens during critical stages of infection. Machine learning predictive tools using natural processing language and transfer learning [23] have leveraged the accuracy of B-cell epitope predictors [24,25] over preceding methods with moderate performance [26]. These advances utilized in combination may enhance prophylaxis against *K. pneumoniae* by finely tuning antigen selection toward high species coverage as well as limiting cross-immunity effects leading to gut dysbiosis.

In this study, we have taken advantage of a recent algorithm and biological data accessibility to find strongly conserved B-cell epitopes in thousands of proteomes of the six principal highly resistant clones. Protein expression, interactivity, and presence of homologs in gut microbiota species were further considered to identify 11 promising universal antigens to prevent *K. pneumoniae* infections in susceptible populations. Finally, we have designed optimal antigen combinations adequate for multivalent formulations with potentially higher and more accurate protective performance.

## 2. Results and Discussion

### 2.1. Evaluation of Published K. pneumoniae RV Studies

We reviewed the literature to identify omic-level RV antecedents utilized for vaccine design against *K. pneumoniae*. We detected five papers using “*Klebsiella pneumoniae*” and “(“reverse vaccinology” or immunoinformatic)” keywords in Pubmed published between 2019 and 2022. Considering its clinical relevance and low vaccine development, these are few RV studies. For example, for other critical pathogens such as *Acinetobacter baumannii*, 11 RV articles were available in 2021 [27].

We selected 24 RV issues, a number of which have been reasonably approached in *K. pneumoniae* studies (Table 1). In general, these strategies were oriented toward the design of multi-epitope hybrid antigens built by peptide linking, as well as structural modeling and molecular dynamic simulation of the resulting polypeptides. These pioneer RV studies mostly resulted in universal antigens for this species, extensible to carbapenem-resistant clones [28,29,30,31,32]. However, other aspects were missing or poorly implemented in the current literature. For instance, no vanguardist B-cell epitope prediction was carried out. Epitope sequence conservation was not profoundly evaluated using thousand-level genome datasets from MDR lineages to anticipate vaccine escape in the actual vaccinable population. Potential interference with the microbiota was weakly approached. The expression pattern and interactomic positioning of antigens were barely considered. Last but not least, no systemic approximation was proposed to find rational antigen combinations that boost the protection of univalent vaccines beyond the empirical merging of top antigens.

In conclusion, several RV issues have been reasonably addressed for *K. pneumoniae*. In contrast, other matters should be further explored in order to enhance our understanding of the *K. pneumoniae* vaccine. These have been attempted below.

### 2.2. Core Proteome of Multidrug Clonal Groups and Exposed Proteins

**Figure 1:**
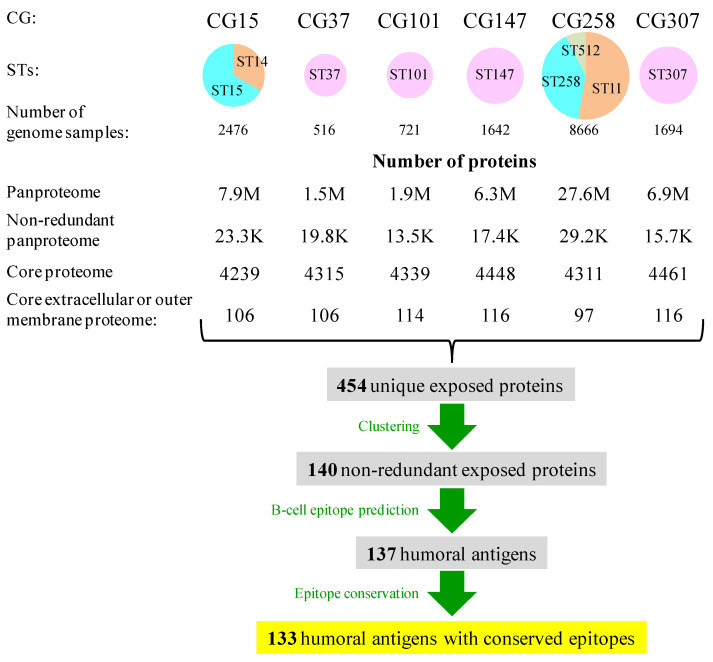
Sizes of genome datasets, pan-proteomes, core proteomes, and exposed proteins in *K. pneumoniae* MDR-CGs. Circles are proportional to the number of genomes and were expressed as sector diagrams when more than one ST was involved to denote the proportion of the genome contribution of each ST to the MDR-CG.

In this study, we focused on the principal MDR lineages that monopolize most MDR intrahospital cases worldwide. These are six CGs, namely CG15 (ST14, ST15), CG37 (ST37), CG101 (ST101), CG147 (ST147), CG258 (ST11, ST258, and ST512), and CG307 (ST307), including nine STs, classified as “problem clones” [9]. In our Spanish Reference Laboratory, these CGs accounted for approximately 75% of all antibiotic-resistant cases [33]. These lineages often carry an array of resistance genes and were named multidrug-resistant CGs (MDR-CGs). Altogether, they were represented by 15,715 NCBI-Assembly genomes (516–8666 range, mean = 2619; ~235 M proteins in total) (Figure 1), which allows for deep intra-clonal analysis. The core proteomes of the six MDR-CGs showed similar sizes (4239–4461, mean = 4349 ± 88 proteins), indicating a strong within-clone genomic redundancy. This is valuable information for detecting pre-existing mutations in epitopes that potentially may lead to vaccine escape.

Immunoprotection against *K. pneumoniae* is mostly humoral [14] and a response, for instance, likely to prevent neonatal sepsis through vaccination of the mother [34]. Searching for antibody-accessible proteins, between 97 and 116 proteins were identified as exposed, either extracellular or in the outer membrane, in the core proteomes of the six MDR-CGs (2.2–2.6% of the respective core proteomes). The aggregation of these searches for all MDR-CGs rendered 454 unique exposed proteins. This became reduced to 140 proteins when clustered at 70% identity and 70% alignment length cutoffs. With these thresholds, proteins within the same cluster were assumed to maintain most epitope residues and domain architectures over more relaxed conditions without significantly affecting the number of representative proteins. More stringent clustering conditions (50% or less identity) only moderately reduced the number of cluster representatives, while more relaxed clustering conditions (e.g., identity = 90%) may divide potential antigens into sub-antigens essentially sharing the same epitopes. In all, 140 non-redundant exposed proteins from at least one MDR-CG core proteome were susceptible to being humoral antigens.

### 2.3. Identification and Intraclonal Conservation of B-Cell Epitopes

The potential capacity of the 140 MDR-CG core exposed proteins described above to elicit humoral prophylaxis was evaluated. For that, continuous B-cell epitopes were predicted with BepiPred 3.0, a transfer learning algorithm from natural processing language. Long-recognized developers of this predictor claimed it was able to capture even discontinuous epitopic traits [24]. The total of 137 (97.9%) MDR-CG core exposed proteins carried epitope spans of at least five residues. This 5mer cutoff was selected as linear runs of five or more residues commonly correspond to complete continuous epitopes or the core section of discontinuous epitopes [35,36]. In total, these 137 antigens accumulated 770 B-cell epitopic zones.

To assess antigenic drift, sequence conservation of these epitopic zones was calculated. This was carried out considering all sequenced samples available of each MDR-CG separately and at the highest per-residue resolution. Although antibody binding shows a range of tolerance to mutations in their respective epitopes [37,38], even minority mutations may anticipate mid-term replacement of present dominant isolates by mutants and subsequent evasion of the immune response elicited by these antigens. Thus, we adopted a conservative approach in which any epitope mutation can invalidate the protection induced by the epitope or reduce its size below the length threshold, as this assumption may favor the selection of broad antibodies. Therefore, a stringent residue conservation threshold of ≥99.5% isolates per MDR-CG was established to permit only occasional sequencing mistakes or transient mutational noise. This step reduced the antigen pool by 2.9% (133 antigens: 40 extracellular and 93 outer membrane proteins) (Table S1) and the total number of epitopic zones by 3.4% (744 epitopic zones, Table S2). Overall, a number of broad epitopes were found, those with sequences extremely conserved in a huge genomic representation of highly resistant clones, which are appropriate for vaccine design.

**Figure 2:**
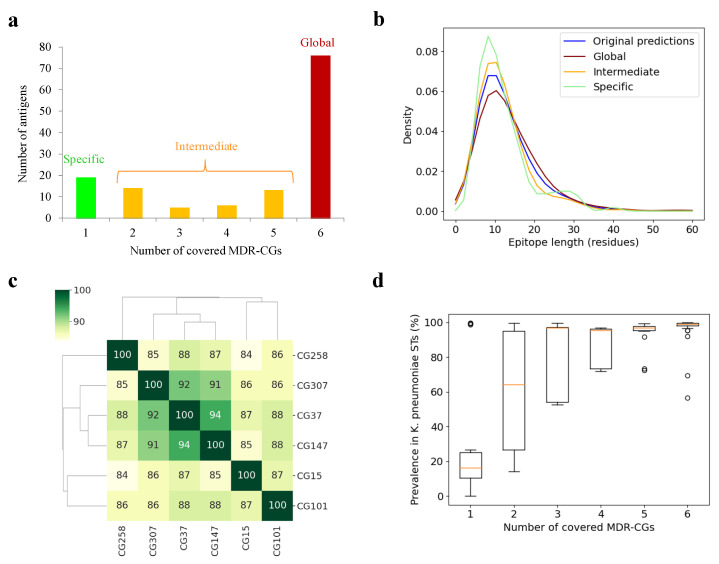
MDR-CG antigens and epitopes. (**a**) Distribution of antigens according to MDR-CG coverage. (**b**) Length distribution of epitopes, including raw predictions by BepiPred3 and those conserved in MDR-CGs. (**c**) Dendrogram and heatmap showing antigen co-existence between all MDR-CG pairs. The percentage of antigenic matching is color-ranked and indicated within the cells. (**d**) Boxplot showing the ST *K. pneumoniae* spread of MDR-CG antigens binned by MDR-CG prevalence. Orange dash indicates the median value. Box limits indicate the interquartile range. Whiskers were adjusted to maximal and minimal values if lower than 1.5 times the IQR. Further outliers are indicated as circles.

As clonal coverage greatly influences their clinical applicability, antigens were classified according to the number of core MDR-CGs in which they were present. This value was assessed using the sum of MDR-CGs that carried any conserved epitope of the antigen. Under our conditions, 76 antigens (57.1% of the total) were represented in the six analyzed lineages and, therefore, were deemed global MDR-CG-global antigens (Figure 2a). At the other end, 19 antigens (14.3%) belonged only to the core of one CG and were termed specific MDR-CG antigens. These ranged from no (CG37) to six specific antigens (CG101). Finally, the remaining 38 antigens (28.6%) were represented between two to five CGs and, thus, were called intermediate MDR-CG antigens. Remarkably, the global MDR-CG antigen pool was enriched in outer membrane proteins (81.2%) with respect to non-global (intermediate or specific) ones (54.4%) (*p* = 0.0005, Fisher exact test), which proportionally contained more extracellular antigens. This indicates antigen location is associated with the degree of clonal coverage.

The epitope length was analyzed in these three coverage bins since, as a result of the application of the sequence conservation cutoff, epitope trimming, splitting, and removal were observed. The raw epitopic zones predicted by BepiPred-3.0 showed an average length of 13.6 ± 11.1 residues, with the bulk of epitopes between 5 and 15 residues (Figure 2b). However, while broad epitopes in intermediate (12.8 ± 10.3 residues) and specific antigens (12.5 ± 6.8 residues) were shorter than the original BepiPred-3.0 predictions, these were longer for global (15.0 ± 14.2 residues) antigen epitopes. Thus, initial epitopes were prone to be split for specific and intermediate antigens, while some epitopes fell below the five residue cutoff and were removed for global antigens. However, this effect was not strong enough to reach statistical significance and did not noticeably alter the percentage of epitopic residues in the whole antigens.

Next, antigen pools of distinct MDR-CGs were compared. This assessment relied on intermediate MDR-CG antigens and may be relevant for the design of high-coverage multivalent vaccines with a safe relationship with the gut microbiota. The antigenic similarity, defined as the percentage of matching antigens, was calculated for all MDR-CG pair combinations (Figure 2c). Remarkably, CG37, CG147, and CG307 shared 91–94% antigens, which suggests these three lineages may be classified as a vaccitype. In contrast, CG15, CG101, and CG258 were more dissimilar, and their prophylactic control may have benefited by clone-specific initiatives.

It should be mentioned that the fact that antigens were classified as MDR-CG intermediate or specific does not necessarily ascertain the protein is fully absent in other MDR-CGs or other *K. pneumoniae* lineages. Some antigens may be widely present in the species but may not satisfy the stringent pharmacological criteria applied here, for clonal membership and epitope conservation, to qualify them for complete CG protection. Indeed, the distribution of antigens within a set of 512 ST-representative *K. pneumoniae* isolates roughly correlated with the number of MDR-CGs represented (Figure 2d, see Section 3). Antigens covering five or six MDR-CGs were present in nearly all *K. pneumoniae* STs. However, the median presence in this *K. pneumoniae* ST-representative pool only decreased below 70% for antigens covering just one or two MDR-CGs, suggesting that those are the ones most adequate for formulations that do not affect commensal *Klebsiellae*.

In all, 133 proteins harboring humoral epitopes with no or negligible sequence drift were detected in the core proteomes of one to six MDR-CGs. Globally distributed antigens are appropriate for universal MDR-CG vaccines, while non-global ones contributed to the definition of MDR-CG vaccitypes and may facilitate precise vaccine interventions.

### 2.4. Potential Cross-Reactions with Host and Human Gut Microbiota

The existence of close immunological homologs of MDR-CG antigens in humans or in their gut microbiota may produce undesirable side effects if included in vaccines. Only five MDR-CG antigens shared significant similarity (E < 0.00001, 21–34% identity over 52–84% antigen length) with human proteins. Moreover, these antigens did not share any identical epitope tract of ≥5 residues with their respective human counterparts. This indicates potential antibody cross-recognition to human proteome by MDR-CG antigen responses is not negligible but, in general, low.

**Figure 3:**
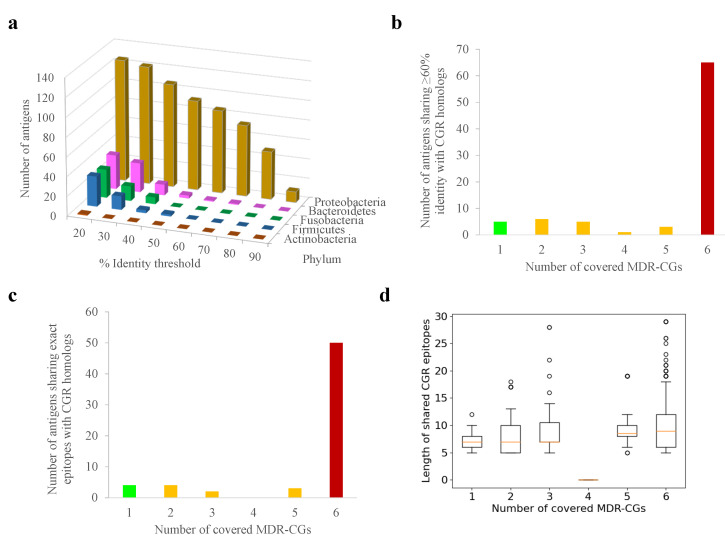
Potential cross-reactivity between *K. pneumoniae* MDR-CG antigens and human gut microbiota proteomes. (**a**) Number of antigens showing homologs with gut microbiota isolates at different sequence identity cutoffs. (**b**) Number of antigens showing homologs with gut microbiota isolates above 60% identity according to MDR-CG coverage. Bars for global, intermediate, and specific MDR-CG antigens are colored in red, orange, and green, respectively. (**c**) Number of antigens sharing epitopes with five residues or with homologs in gut microbiota isolates according to MDR-CG coverage. Bars for global, intermediate, and specific MDR-CG antigens are colored in red, orange, and green, respectively. (**d**) Boxplot showing length distribution of MDR-CG epitopes shared with human gut microbiota. Values were organized by the number of MDR-CGs represented by the epitope. Orange dash indicates the median value. Box limits indicate the interquartile range. Whiskers were adjusted to maximal and minimal values if lower than 1.5 times the IQR. Further outliers are indicated as circles.

A similar analysis was conducted with the human gut microbiota, which may be altered by cross-reactivity, causing health problems. In contrast to the human proteome, nearly all MDR-CG antigens (125, 94.0%) showed one or more homologs in gut microbiota isolates stored in the general CGR resource, at least when relaxed thresholds (E < 0.00001, ≥50% antigen length) were applied (Figure 3a). Among these, homologs from *Proteobacteria* isolates (*Enterobacter*, *Escherichia*, and *Citrobacter* species) dominated in terms of number and identity level. Nevertheless, 50 antigens (37.6%) still had homologs in non-*Proteobacteria* phyla, and 31 of them (23.3%) even in gram-positive phyla (*Actinobacteria* and *Firmicutes*). Up to 85 MDR-CG *K. pneumoniae* antigens (63.9%) still had gut microbiota homologs sharing 60% identity or more with CGR isolates, which are those that, in practice, may elicit cross-reactive antibodies. These antigens were relatively enriched in global MDR-CGs (76.5% of the total, *p* < 0.0001, Fisher exact test) (Figure 3b) compared to intermediate or specific ones. Microbiota homologs over 60% identity were only detected in *Proteobacteria* (Figure 3a), except the lipoprotein Blc also present in *Bacteroidetes*.

When exact 5mer epitopes or longer were searched in gut microbiota homologs, the incidence of *Proteobacteria* was exclusive. Global MDR-CG antigens shared proportionally more conserved epitopes (73.7%) than intermediate and specific antigens, including identical epitopes with 15 or more residues (76.6%) (Figure 3c). Remarkably, only 11 global MDR-CG antigens satisfied the confronted forces of covering all MDR-CGs but were not represented (less than 60% identity and no epitopes) in any of the gut microbiota isolates.

Overall, the risk of immunological cross-reactivity after vaccination with MDR-CG antigens is emphasized in relation to global antigens and *Proteobacteria*. In any case, precise cutoffs regarding the degree of homolog closeness in the gut microbiota can be implemented in RV procedures for antigen selection. This would allow us to find a balance by finely tuning sensitivity (protection) and specificity (lack of cross-reactions) when generating antigen candidate lists.

### 2.5. Identification of Antigens with High Functional Constraints

**Figure 4:**
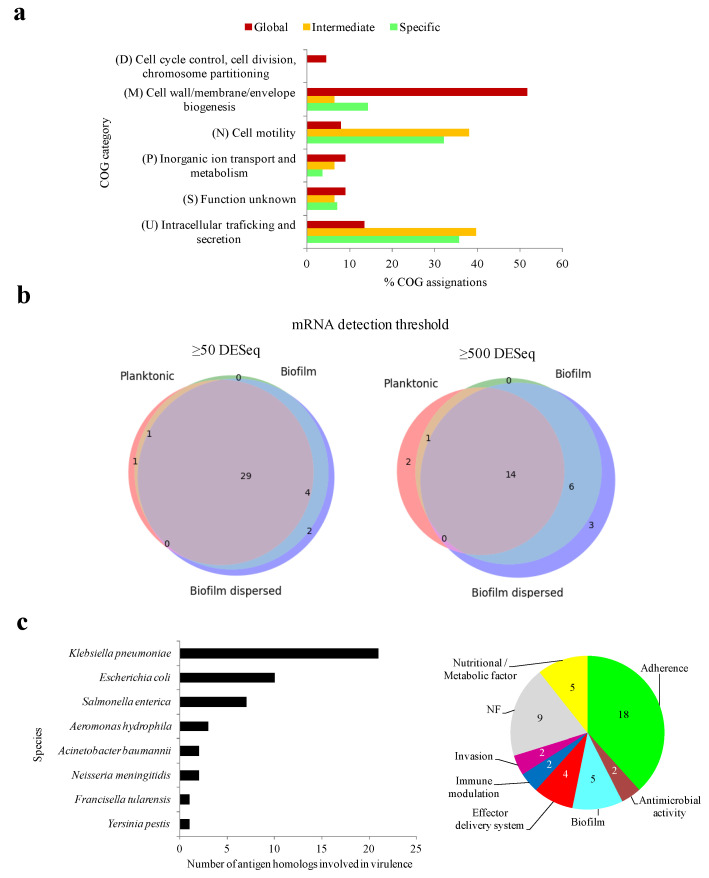
Functional constraints of MDR-CG antigens. (**a**) General functional category of antigens according to MDR-CG coverage. Only categories with at least 4% COG assignation in a MDR-CG coverage bin were included. (**b**) Weighted three-class Venn diagrams showing the number of expressed antigens during planktonic, biofilm, and biofilm dispersed stages. Low (≥50 DESeq, **left**) and high (≥500 DESeq, **right**) RNA-Seq detection thresholds were considered. The Venn diagrams were depicted using the *Venn_3* method of the *matplotlib* Python library. (**c**) Species origin of putative virulence antigens (**left**). Prevalence of VF categories for antigens (**right**).

Certain functional aspects of the antigens may be associated with evolutionary constraints that reduce the chances of epitope drift, shift, or loss. Regarding general functional classification, global MDR-CG antigens were proportionally enriched in housekeeping-like functions concerning the “Cell wall/membrane/envelope biogenesis” class, while intermediate and specific antigens were more often classified within “Cell motility” and “Intracellular trafficking and secretion” categories (Figure 4a). This functional swap may have implications when precise immunoprotection is designed.

Knowing the expression regime of an antigen is crucial for its ultimate utilization. To address this issue, transcriptomic data previously published from five growth stages, including distinct nutrient availability and environmental conditions, were utilized [39]. Equivalent proteins for 108 out of 133 antigens (81.2%) were detected in the strain CH1034, the one utilized in the original study. From them, transcription for 37 and 26 (27.8% and 19.5%) MDR-CG antigen homologs was observed in at least one condition after applying cutoffs of 50 and 500 DESeq normalized values and deemed expression and high expression, respectively. Up to 31 antigens were expressed in both planktonic conditions (exponential and stationary), 49 in the two biofilm conditions (biofilms of 7 and 13 h), and 54 during the “biofilm dispersed” stage. Expression for 29 antigens was detected during all conditions (Figure 4b, left), and 14 of them were always expressed at high counts and were, therefore, deemed constitutively highly transcribed antigens (Figure 4b, right). The last group included 12 outer membrane proteins, of which five belonged to the “Cell wall/membrane/envelope biogenesis” and four to the “Inorganic ion transport and metabolism” COG categories.

Next, we studied whether antibodies elicited by the MDR-CG antigens may ameliorate infection through direct neutralization of virulent activities. Forty-seven antigens (35.3%) shared significant homology with virulence factors (VFs). Almost half of them, 21, were genuine *K. pneumoniae* VFs while the remaining were mostly homologs to VFs from other *Enterobacteria* pathogens such as *E. coli* and *Salmonella enterica* subsp. enterica serovar Typhimurium (Figure 4c, left). Eighteen antigens were classified within the “Adherence” category, five were in the “Nutritional/Metabolic factors” category, and five were classified in the “biofilm” virulence category (Figure 4c, right). Thus, the humoral response against one-third of the MDR-CG antigens may directly interfere with several pathogenic mechanisms of *K. pneumoniae*.

**Figure 5:**
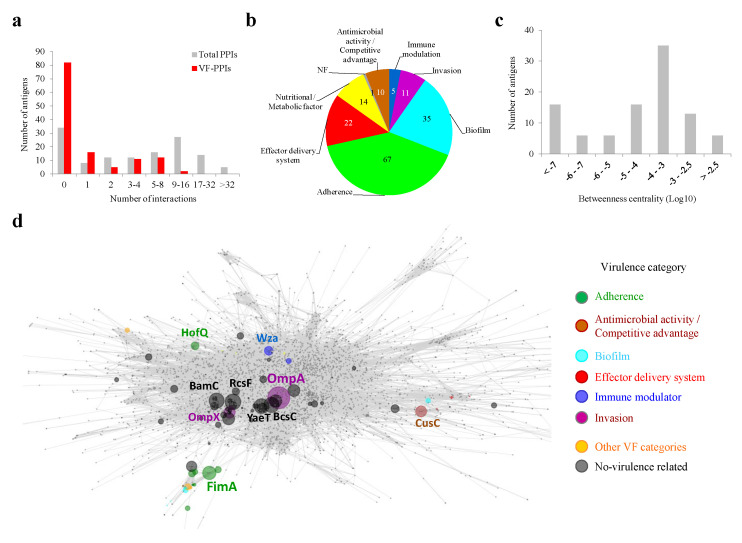
Interactomic properties of MDR-CG antigens. (**a**) Antigen distribution according to the total number of PPIs and VF-PPIs. (**b**) Prevalence of VF categories for first-rank interactions of antigens. The cumulative number of PPIs, i.e., one protein can interact with several antigen partners, is shown. (**c**) Antigen distribution according to BC values. (**d**) Topology of the *K. pneumoniae* interacting network. Antigens are colored by virulence categories if applicable. The sphere diameter of antigen nodes is proportional to BC value. Antigen nodes are name-labeled when above a BC cutoff of 0.3% or 0.1% if they are VFs. Non-antigen nodes are shown as small dots in gray. Edges are shown in gray.

Finally, the influence of MDR-CG antigens on the *K. pneumoniae* network of protein-protein interactions (PPIs), the interactome, was analyzed since the types and extent of the connections of a protein correlate with its global relevance on cellular physiology [40]. For that, we applied the principles of network theory, by which proteins are converted into nodes, and their interactions are taken as edges. Equivalent proteins for 101 out of the 133 antigens (75.9%) were identified in HS11286, the *K. pneumoniae* reference strain of the central interactome resource STRING. From those, 90 antigens showed interactions with other *K. pneumoniae* proteins, ranging from 1 to 77 PPIs (Figure 5a). In addition, 46 antigens showed first-rank PPIs to at least one virulence factor (VF-PPIs), suggesting potential indirect neutralization influence of their elicited antibodies. For VF partners interacting with antigens, “Adherence” was also the category that was more prominent (Figure 5b). Therefore, it is possible that the neutralization of this pathogenesis stage through vaccination could be further amplified through functional contacts of antigens with adhesive proteins.

Among the principal virulence hub antigens, two Hcp1 homologs from Type VI secretion systems excelled with 11 and 10 VF-PPIs with “Effector delivery system” proteins. Furthermore, two FimD fimbrial proteins exhibited eight interactions, each involving biofilm and adherence factors. An interactome metric of protein importance over the raw PPI count is BC (see Section 3). BC values of antigens followed a rather normal distribution, with a peak between 10^−3^ and 10^−4^ (Figure 5c). Six antigens showed a betweenness centrality over 0.3% shortest paths (labeled with protein name in the network graph depicted in Figure 5d), including the invasion-related protein OmpA (BC = 0.96%). Thus, there are MDR-CG antigens that show an attractive positioning in the topology network for magnified neutralization of *K. pneumoniae.*

### 2.6. Selection for Global and Constitutive Expressed Antigens for Univalent Vaccines

**Figure 6:**
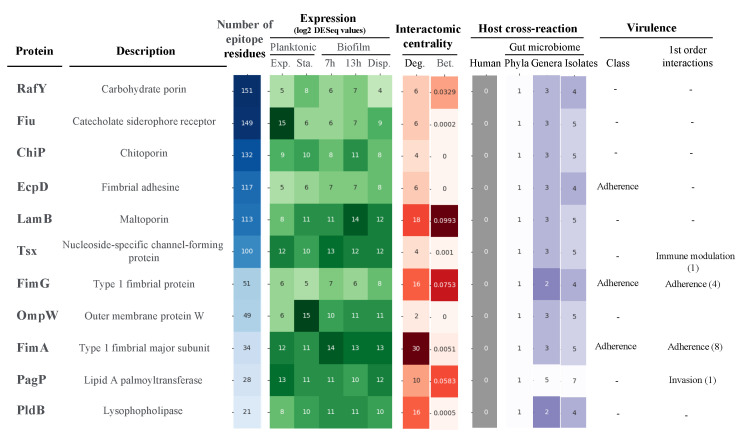
Antigens selected for univalent vaccines against *K. pneumoniae* MDR-CGs. Antigens were inversely sorted by the raw number of conserved B-cell epitope residues. Values for filtering criteria were color-ranked where the intensity was proportional to the positive condition. The number of first-order interactive partners grouped by virulence class is provided in brackets.

Despite the evident properties of antigen prioritization, no unique selection scheme can be considered. To dynamically check different selection scenarios, all data generated above were normalized as a single framework. In a first approximation, we studied proteins separately for univalent vaccines. For this, we focused on the clonal scope and conserved epitopicity over the six principal MDR lineages, as well as constitutive expression. Moreover, we established these antigens should show modest conflict with the gut microbiota, i.e., only *Proteobacteria* homologs at most, as these anaerobic, facultative species account for less than 0.1% of bacteria in the oxygen-deprived colon environment [41]. Out of the initial 76 global MDR-CG proteins, only 13 satisfied all these criteria, including several porins and fimbriae. Importantly, this list was further reduced to 11 antigens, as shown in Figure 6, because the BamD and BamE outer membrane assembly proteins were actually oriented inwards of the cell and, thus, were not accessible to antibodies [42]. This indicates that, in conjunction with computational methods, exhaustive manual analyses by human experts are ultimately required to evaluate RV outcomes prior to labor-intensive experimental validation. Notably, vaccination with any of the three extracellular proteins in this subset (EcpD, FimA, and FimG) would predictably elicit virulence neutralization. Likewise, four out of the 11 proteins showed BC values above 0.01% of the shortest paths. Antigens in this list essentially showed attractive experimental predicted solubility, non-toxic, and non-allergenic properties (Table S3). Altogether, these 11 antigens are attractive candidates for univalent approaches for either protein or nucleic acid vaccines.

### 2.7. Antigen Selection for Multivalent MDR K. pneumoniae Vaccines

Multivalent vaccines achieve high efficacy by limiting potential escape and low coverage, often shown by univalent formulations [43]. Therefore, we studied different rational antigen combinations, taking advantage of the harmonized data described above. Bivalent to pentavalent options were obtained based on the selection reasoning.

We designed a first approach consisting of highly epitopic proteins (>100 B-cell epitope residues). Moreover, among these antigens, those showing high expression certainty in at least one infection stage were chosen. This was based on the assumption that to reach optimal results with nosocomial pathogens, it is necessary to combine antigens expressed during planktonic and biofilm stages [44]. We established the microbiota cross-reactivity and BC filters to two phyla/seven genera and 10^−6^ shortest paths, respectively. These criteria resulted in a trivalent combination of the “catecholate siderophore receptor” Fiu (high “exponential planktonic” transcription), the “maltose and maltodextrins transporter” LamB (high “stationary planktonic” and “late biofilm” expression counts) and the “nucleoside-specific channel-forming protein” Tsx (high “biofilm expression” levels). Remarkably, these three proteins are involved in the acquisition of different key nutrients, which may be simultaneously undermined by the humoral protection elicited.

Next, direct neutralization of pathogenicity was analyzed as antibodies against VFs may behave as an “anti-virulence” defense [45]. In this context, two antigen selection paradigms are possible for the VF antigens utilized belonging to a single or several virulence categories. The aims are magnifying the focus on an event or avoiding functional redundancy, respectively. An illustrative example is adhesion, the most predominant activity to be neutralized through vaccination. However, the fact that numerous antigens exert this function, clones that eventually delete adherent-focus antigens to escape the vaccine may remain infective. Nevertheless, a mixture including the tip subunits of Fim and LpfC fimbria, as well as the PilQ porin, may be considered for preliminary assays. Virulence categories less prone to functional overlapping were “biofilm formation” and “Invasion”, which are the other two critical phases during *K. pneumoniae* infection. A bivalent option that may elicit immunological interference of biofilm formation consist of the efflux pump AdeH and MrkC, the latter being the most attractive subunit of the Mrk fimbria by epitopicity, expressability, and network centrality criteria. Invasion may be hindered by immunization with the OmpA porin and the Ail evasin.

**Figure 7:**
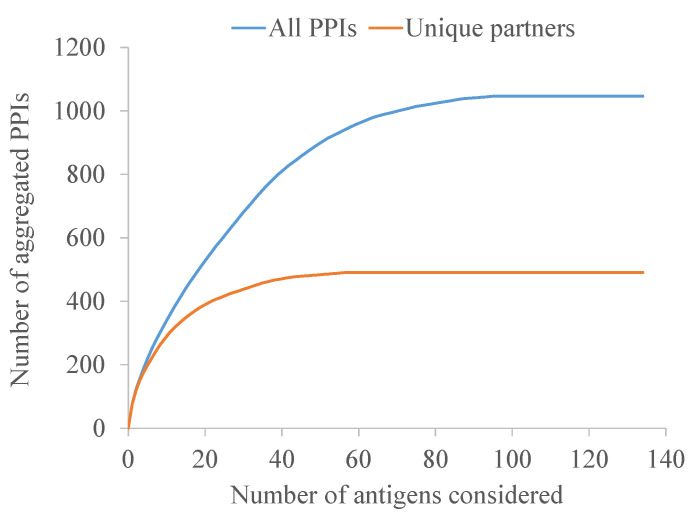
Aggregated number of all PPIs and those with non-redundant partners of antigens. Antigens were reversely sorted on the x-axis by their contribution to the interactome.

Finally, a systems biology approach was conducted to maximize neutralization through the synergy of non-related interactomic hubs. The 98 antigens with predicted PPIs, after excluding BamCDE, accounted for a total of 1025 PPIs involving 491 unique protein partners. When the MDR-CG antigens were sorted greatest-to-least by the number of PPIs, a plateau was reached much earlier when only the non-redundant interacting proteins were considered (Figure 7). In fact, a collection of just five antigens, namely OmpA, OmpX, BcsC, BamA, and Hcp amounted to 41% of all unique partners. Thus, antibody-driven neutralization through this pentavalent formulation may be amplified through functional interactions to produce a vast disturbance in the protein network architecture and, subsequently, in the cell physiology.

Altogether, rational antigen combinations concerning high epitopicity, virulent categories, and interactomic warrant experimental validation to gain insights into the most successful strategies in practice.

### 2.8. The Combination of Intermediate MDR-CG Antigens

**Figure 8:**
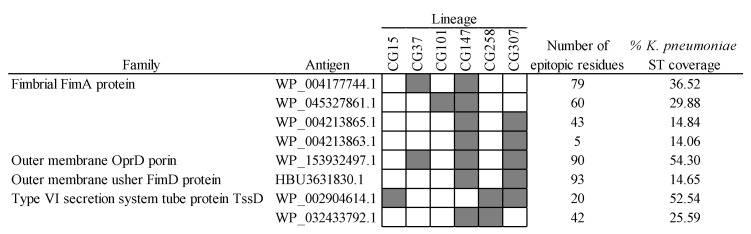
Intermediate MDR-CG antigens showing low gut microbiota and low general *K. pneumoniae* ST predicted cross-reactivities. Representation (filled cells) and non-representation (empty cells) of the MDR-CGs by the antigen are indicated.

Another promising combinative approach to produce effective vaccines relies on the fine selection of complementary antigens with intermediate MDR-CG coverage. The advantage of doing this is that cross-reactive side effects may be reduced while maintaining protection against highly resistant clones. Out of the 38 intermediate MDR-CG antigens, 8 (6 were extracellular, and 5 were VFs) were not represented in microbiota phyla other than *Proteobacteria* and in ≤60% of *K. pneumoniae* ST representatives (Figure 8). These antigens involved four homologs of FimA fimbrial protein, two Type VI secretion system tube TssD proteins, one usher fimbrial protein, and one OprD porin. Despite the obvious relationship of homologs belonging to either FimA or TssD families, they only shared moderate identities of ≤30% and 61% with other members of the same family, respectively, which further decreased to ≤14% and 50% identities in the epitopic regions. This supports intermediate MDR-CG antigens with comparable functions that are immunologically independent and adequate for co-selection in the same formulation. Moreover, wide MDR-CG protection may be achieved by adding antigens that cover combos of two, three, or four lineages while maintaining microbiota safety profiles. This combinatorial plan was congruent with the vaccitype paradigm depicted above (Figure 2c), as the CG147-CG307 clonal pair shared up to four of these antigens. Moreover, these two CGs were abundantly considered by this type of strategy (seven and five intermediate antigens, respectively). In contrast, CG101 only had one of these intermediate antigens, which likely required the addition of CG101-specific ones (see below) to obtain sufficient clonal coverage. As a result of this analysis, a trivalent intermediate antigen vaccine with a fimbrial FimA homolog (WP_045327861.1), a TssD homolog (WP_002904614.1), and the only porin (OprD), would cover the six MDR-CGs with high specificity.

### 2.9. The Utilization of Specific MDR-CG Antigens

**Figure 9:**
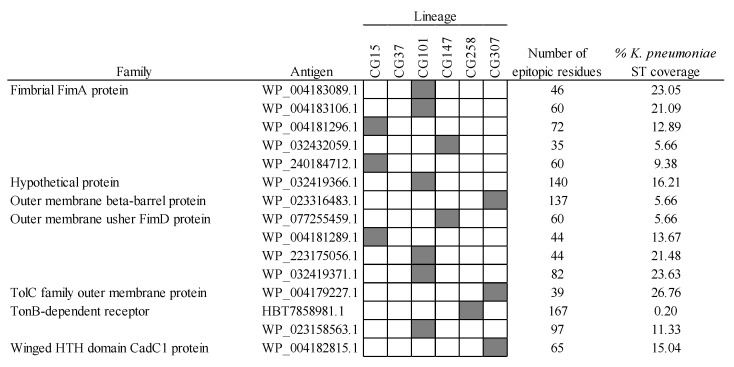
Specific MDR-CG antigens showing low gut microbiota and low general *K. pneumoniae* ST predicted cross-reactivity. Representation (filled cells) and non-representation (empty cells) of the MDR-CGs by the antigen are indicated.

Finally, another precise vaccinable strategy consists of the utilization of specific MDR-CG antigens, which may even further reduce the interference with microbiota and commensal *K. pneumoniae* isolates. Out of the 19 MDR-CG-specific antigens, 15 showed extraordinary microbiota safety profiles. In some cases, homologs were detected in ≤1 microbiota *Proteobacteria* isolates and were present in <30% *K. pneumoniae* representative STs (Figure 9). The 15 reliably specific antigens showed epitopicities ranging from 39 to 163 MDR-CG conserved residues, suggesting a sufficient antigenic capacity. All CGs but CG37 had at least one CG-specific antigen, while CG101 accounted for six.

Functional description for nearly all of these antigens was obtained, which is important to rationalize the mode-of-action of elicited antibodies, i.e., neutralization, over empirical protection. Remarkably, nine antigens from four MDR-CGs were annotated as fimbriae subunits, which, like for intermediate MDR-CG fimbria, had low chances of inducing cross-protection. Seven of these fimbrial antigens were phylogenetically closer to adherent virulence factors from isolates of other enterobacterial pathogens. This suggests these particular antigens were acquired by lateral transfer at *K. pneumoniae* post-speciation stages and maintained through improving the colonization abilities of the resulting clone. Thus, tissue-specific adhesion of CG15, CG101, and CG147 may be approached with high preciseness inducing neutralizing IgA antibodies against their hallmark fimbriae.

Despite reasonable epitopicity of intermediate (50.0 ± 28.5, average ± SD, epitopic residues) and specific (77.6 ± 39.5) MDR-CG antigens, an obvious disadvantage for these approaches is no extensive information, e.g., expression or interacting data, can be inferred for most of these proteins due to lack of representation in working strains. Furthermore, a prohibitive number of antigens must be utilized to achieve full MDR-CG protection. Nevertheless, they may still be considered to complement the intermediate MDR-CG antigen combinations. Moreover, epitopic zones of specific antigens may be selected for peptide-based vaccines or for the construction of hybrid antigens based on loop grafting into scaffold proteins [46]. Importantly, one intermediate and one specific antigen (Type VI secretion system tube TssD protein and TonB-dependent receptor homologs, respectively) were identified for CG258, which is still the most challenging high-resistant clone worldwide. Compared with global MDR-CG antigens, the use of specific MDR-CG antigens embodies the ideal of “magic bullet” vaccination that mostly circumvents cross-reactivity with health-related commensal *Proteobacteria* strains as those concerning K vitamin production and prevention of colonization by pathogens [47]. This strategy may be useful for hospitals with recurrent infections by the same endemic clone [48,49], in particular for patients prone to microbiota alterations.

## 3. Materials and Methods

### 3.1. Sequence Acquisition and Clustering

Non-redundant complete proteomes of *K. pneumoniae* from the Genbank and RefSeq databases were acquired from the NCBI-Assembly resource [50]. Samples that were non-annotated, partial, anomalous, and those that did not pass the taxonomy check were excluded. Isolates were assigned to ST by applying BLAST 2.2.26, with strict 100% identity and 100% alignment coverage, on whole genomes using sequence types (STs) gene allelic combinations and sequences stored at the MLST database (https://pubmlst.org/) (accessed on 27 March 2023) [51]. Proteins were clustered with CD-HIT 4.6 [52] at 70% identity and 70% alignment length. Proteins showing homologs over these thresholds in ≥95% isolates from a given clonal group (CG) by BLAST were deemed core proteins of that CG.

### 3.2. Sequence Analyses

Among all CG core proteins, those exposed (extracellular or outer membrane cellular locations) were identified using PSORTb 3.0.3 by selecting the “Gram-negative” option [53]. Exposed core proteins from any CG of interest were pooled and subjected to further clustering with CD-HIT applying a 70% identity and 70% alignment length. B-cell linear epitopes were predicted with BepiPred 3.0 [24], applying the default threshold for the rolling mean score. Predicted B-cell epitope spans with lengths ≥ 5 positions were subjected to residue-level conservation analysis within the CG with Clustal Omega 1.2.4 [54]. Only those epitope regions with five or more residues that were strictly conserved in ≥99.5% isolates within the CG were considered broad epitopes and considered for further analyses. To assign the sequence limits of broad epitopes, the highest number of CGs was prioritized over epitope length even when its sequence was reduced by trimming or splitting due to non-conserved positions in any of the CGs, providing epitopes were five residues or longer. For this process, an in-house Python script was programmed. The final MDR-CG coverage of an antigen was defined as the total number of MDR-CGs in which at least one broad epitope of the antigen is present.

Membership in the *K. pneumoniae* core proteome sensu lato (https://www.cgmlst.org/ncs/schema/Kpneumoniae865/) (accessed on 4 August 2023) was determined with MMSeqs2 v.13-45111+ds-2 applying 70% identity and 70% alignment length thresholds. The global occurrence of antigens in the whole *K. pneumoniae* species was estimated by selecting representative samples of 512 STs with five or more sequenced samples in NCBI-Assembly (Table S4) using MMSeqs2 [55] applying 70% identity and 70% alignment length coverage as cutoffs.

Functional categories according to the Cluster of Orthologous Groups (COGs) scheme were assigned with eggNOGmapper v2 [56] against the eggNOG 5 database [57] using default parameters. Protein domains in the Pfam v33.1 database [58] were detected with hmmscan of HMMER 3.1b1 [59] applying score gathering thresholds.

### 3.3. Host and Human Gut Microbiota Homologs

For the detection of antigen homologs in the human proteome, the reviewed *Homo sapiens* SwissProt proteome downloaded from UniProt was used (accessed 29 March 2023). The global presence of antigen homologs in the gut microbiota was assessed using representative genomes covering culturable gut microbiota stored in the Culturable Genome Reference (CGR) archive [60]. CGR contains 306 bacterial isolates stratified into 176 species, 50 genera, and 5 phyla. For both human and CGR proteomes, antigen sequence searches were carried out with MMSeqs2 [55], applying different identity, alignment coverage, or E-value cutoffs. Exact *K. pneumoniae* epitope sequences with five or more residues present in CGR and human proteomes were identified with an in-house Python 3.10.12 script. The only *K. pneumoniae* representative isolates in the CGR was not considered in gut microbiota analyses.

### 3.4. Expression, Virulence, and Interactome Data

Normalized DESeq transcriptomic expression data from exponential (ExPlan) and stationary (StatPlan) planktonic cells, cells after 7 h (7hBiof) and 13 h (13hBiof) biofilm growth, and cells dispersed from biofilms (DispBiof) were acquired from previously published data [39]. Antigen homologs in the original strain of this study, CH1034 (NCBI-Assembly id: GCF_001404095), were identified by search with MMSeq2 applying thresholds of 70% identity and 70% alignment length. Different cutoffs for normalized DESeq values were applied to consider expression.

To detect putative proteins involved in virulence, antigens were compared with the set A content of the virulence factor database (VFDB) using MMSeq2 and applying thresholds of 30% identity and 60% alignment length.

For interactomic analyses, homologs for antigens in the *K. pneumoniae* representative strain available in STRING v11 [61], HS11286, were identified by search with MMSeq2, applying thresholds of 70% identity and 70% alignment coverage. Then, pre-calculated edges showing a combined score of 0.7 or higher (“Confident level”) were selected. Betweenness centrality (BC) was calculated based on these data using the betweenness_centrality method of the NetworkX 3.0 Python package [62]. BC is defined as the fraction of shortest paths between all node pairs in which an antigen node is included.

### 3.5. Calculation of Antigen Workability, Allergenicity and Toxicity

Solubility of overexpressed proteins in *Escherichia coli* was assessed with SoluProt 1.0 [63], toxicity with the hybrid model of ToxinPred2 [64], allergenicity with AllerCatPro 2.0 [65] and transmembrane helices with Phobius [66]. All these predictions were carried out after removing the signal peptide when detected with SignalP 6.0 using the non-eukaryotic ‘other’ model [67].

## 4. Conclusions

In this study, we have applied several antigen selection criteria not considered previously for K. pneumoniae vaccination. For instance, the conservation assessment of epitope sequences using massive genomic data per CG allowed us to enter immunoinformatics into the deep intraclonal age. Consequently, this allowed us to detect pre-existing minority subclones with mutated epitopes that may eventually lead to vaccine escape. Likewise, antigen- and epitope-level conservation analyses in a comprehensive list of gut microbiota taxons rendered a more precise estimation of potential undesirable cross-reactions. In contrast to previous *K. pneumoniae* RV reports, essentiality was not applied here because, unlike antibacterial targets, many vaccine antigens are only indispensable for optimal fitness. Our analyses permitted a most confident estimation of antigen epitopicity and the protective clonal scope for each antigen and revealed a potential MDR vaccitype that included CG37, CG147, and CG307 clades.

Humoral antigens showed a range of clonal coverage, microbiota homologs, number of epitope residues, expression conditions, functions, and interactions. A number of them were directly or indirectly related to adhesion during infection. Of the 133 antigens represented in the MDR lineages, only 11 satisfied most of the general criteria and were deemed promising for univalent vaccines. Remarkably, manual inspection revealed some of the original antigens were very likely not accessible to antibodies, which suggests RV outcomes must be carefully revised by experts. Furthermore, several strategies were utilized concerning clonal coverage, expression, virulence, and positioning in the protein network. For instance, as few as five antigens accumulated 41% of all non-redundant partners in the interactome of conserved-epitope antigens. The reasoned utilization of antigens with middle and exclusive clonal MDR coverage increased the preciseness. Notably, among other functionalities, many promising antigens consisted of porins and fimbriae.

In sum, the insights obtained here provide important clues that will facilitate the design of improved vaccines against this primary human pathogen, in particular for synergic reasoned multivalent and low vaccine-escape approaches.

## Figures and Tables

**Table 1:** Summary of RV antecedent reports for *K. pneumoniae*. NA: non-applied.

RV Items	Dar et al., 2019 [28]	Mehmood et al., 2020 [29]	Allemailem, 2021 [32]	Wang et al., 2022 [31]	Cuscino et al., 2022 [30]	This Study
INPUT DATA						
Number of genomes considered	222	173	973	274	467	15,715
Protein identity threshold	90%	50%	NA	Default roary	NA	70%
Clinical phenotype	NA	NA	NA	Prevalent capsular types	467 carbapenem-resistant isolates	The six most prevalent MDR lineages
Antigen clonal prevalence	NA	NA	NA	NA	95% isolates	95% isolates within CGs
Deep intraclonal analysis	NA	NA	NA	NA	NA	Yes (516–8666 genomes per CG)
Protein location	PSORTb 2.0	PSORTb 3.0	PSORTb 3.0	PSORTb 3.0	PSORTdb 4.0	PSORTb 3.0.3
EPITOPE ANALYSIS						
Prediction of B-cell epitopes	ABCPred	ABCPred	BepiPred 2.0	ABCpred	ABCpred	BepiPred 3.0
Epitope coverage	NA	NA	NA	NA	>80% identity (full protein)	Epitope conservation in CGs
Epitope sequence conservation	NA	NA	NA	NA	NA	Per residue within CG
Prediction of HLA II epitopes	ProPred/MHCPred 2.0	BcePred	MHCPred 2.0	IEDB server	NetMHCIIpan-3.2	NA
SIDE EFFECTS						
Homology with human proteins	Whole antigen BLAST	Whole antigen BLAST	NA	NA	NA	Whole antigen and epitope MMSeqs2
Homology with gut microbiota	NA	GFDB	*Lactobacillus* spp.	NA	*E. coli* K-12	Three-level stratified taxons (307 representative genomes). Whole protein and epitope searching
Toxicity	NA	NA	ToxinPred	NA	NA	ToxinPred
Allergenicity	AllergenFP 1.0; AllerTOP 2.0	NA	AllerTOP 2.0	AllergenFP 1.0; AllerTOP 2.0	AllergenFP 1.0; AllerTOP 2.	AllerCatPro 2.0
FUNCTIONAL CONSTRAINTS						
Antigen expression	NA	NA	NA	NA	NA	5 conditions
Virulence	MvirDB; VFDB	VFDB	VFDB; VirulentPred; SPAAN	NA	VirulentPred	VFDB
Interactomics	NA	NA	NA	NA	NA	Centrality from STRING data
Essentiality	DEG	DEG	NA	NA	NA	NA
WORKABILITY						
In vitro solubility	SOLpro; PROSO II	NA	NA	NA	Protein-sol	SoluProt
Transmembrane helices	HMMTOP 2.0; TMHMM 2.0	NA	NA	NA	NA	Phobius
PROPOSED VACCINE						
Molecular dynamics	GROMACS	NA	AMBER20	GROMACS	GROMACS	NA
Antigen type	NA	NA	Peptides	Epitopes	Epitopes	Antigen combinations
Predicted antigenicity	ANTIGENpro; VaxiJen v.2.0	VaxiJen v.2.0	NA	VaxiJen	ANTIGENpro; VaxiJen 2.0	NA
Criteria for synergic multivalency	NA	NA	NA	NA	NA	Several

## Data Availability

All relevant data are included in the manuscript and its supporting information files.

## Supplementary Materials

The supporting information can be downloaded at: https://www.mdpi.com/article/10.3390/ijms25189837/s1.
